# Diffused calcification in a patient with long-term warfarin therapy: a case report

**DOI:** 10.1093/ehjcr/ytac364

**Published:** 2022-09-01

**Authors:** Bryan Richard Sasmita, Suxin Luo, Bi Huang

**Affiliations:** Department of Cardiology, The First Affiliated Hospital of Chongqing Medical University, Chongqing 400016, China; Department of Cardiology, The First Affiliated Hospital of Chongqing Medical University, Chongqing 400016, China; Department of Cardiology, The First Affiliated Hospital of Chongqing Medical University, Chongqing 400016, China

**Keywords:** Warfarin, Vascular calcification, Tracheobronchial calcification, Valvular calcification, Anticoagulant, Case report

## Abstract

**Background:**

Lifelong warfarin is mandatory in patients with mechanic valvular replacement. The main adverse effect of warfarin is haemorrhage; however, there are several rare adverse events associated with long-term warfarin treatment, such as calcification, cholesterol microembolization, and nephropathy. Here we report a case of chronic warfarin use that gradually manifested with diffused calcification.

**Case summary:**

A 78-year-old woman received a prosthetic mechanical mitral valve replacement when she was 46 years old due to rheumatic mitral stenosis. She has been taking warfarin ever since. Ten years prior to admission, the chest radiography revealed a mild diffused calcification tracheobronchial and subsequent chest imaging indicated a progressive calcification of the tracheobronchial tree. In addition, a series of echocardiography examinations indicated progressive calcific aortic stenosis and diffused calcification in abdominal aorta. Furthermore, the patient gradually presented with advanced heart failure. Finally, she received transcatheter aortic valve replacement and the symptoms of the heart failure significantly improved.

**Discussion:**

Currently, patients with valvular atrial fibrillation or mechanical valve replacement have no other choice for anticoagulation medication except warfarin. However, long-term use of warfarin was associated with some rare complications such as diffused calcification. Therefore, close monitoring of such side effects in patients with long-term use of warfarin is warranted.

**Learning points**

- Long-term use of warfarin is recommended for patients with valvular atrial fibrillation or mechanical valve replacement. However, some complications associated with long-term use of warfarin may happen, such as diffused calcification in valve, aorta, and bronchial tree.
- Calcification induced by long-term use of warfarin is associated with the loss of the protection provided by matrix gamma-carboxyglutamate Gla protein (MGP). Therefore, warfarin leads to decreased MGP and induces vascular calcification.
- Tracheobronchial calcification induced by warfarin is usually asymptomatic and is not a contraindication for continuing warfarin therapy. In contrast, if calcification occurs in the valve or the main artery and causes severe stenosis, it is an indication of further therapy.

## Introduction

Warfarin is an oral anticoagulant that works by competitively inhibiting vitamin K epoxide reductase complex 1 (VKORC1) which is an essential enzyme for activating vitamin K, thus interfering synthesis of several clotting factors, such as Factors II, VII, IX, and X, as well as coagulation regulatory factors Protein C and Protein S. Warfarin is commonly used to treat and prevent thromboembolic disorders; however, long-term use of warfarin was associated with some rare complications. Here we report a case presented with diffused calcification, possibly associated with long-term prophylactic anticoagulant therapy with warfarin after prosthetic mechanical mitral valve replacement.

## Timeline

**Table:** 

Time	Event
32 years prior	The patient received a tilting disc mechanical mitral valve (Size 26#, Shanghai made) due to rheumatic mitral stenosis.
16 years prior	Routine chest radiography and cardiac echocardiography were unremarkable.
10 years prior	Chest radiography indicated a mild diffused tracheobronchial calcification.
5 years prior	Cardiac echocardiography reported a severe calcific aortic stenosis. Chest computed tomography (CT) demonstrated an extensive calcification of tracheobronchial rings. The patient was suggested to receive a valvular replacement surgery, but she refused the intervention.
May 2021 (Admission time)	The patient dyspnoea became worse and got readmitted. Chest CT showed progressive calcification of the tracheobronchial ring compared to 5 years ago. Echocardiographic examination revealed severe aortic stenosis with a valve orifice of 0.5 cm^2^. CT angiography demonstrated diffused calcification in the abdominal aorta and mild calcification in the left anterior descending artery. The patient received a transcatheter aortic valve replacement (Size L23, Venus, MedTech).
6 months follow up	The patient remained free of exertional dyspnoea and oedema of the lower limbs.

## Case presentation

**Figure 1:**
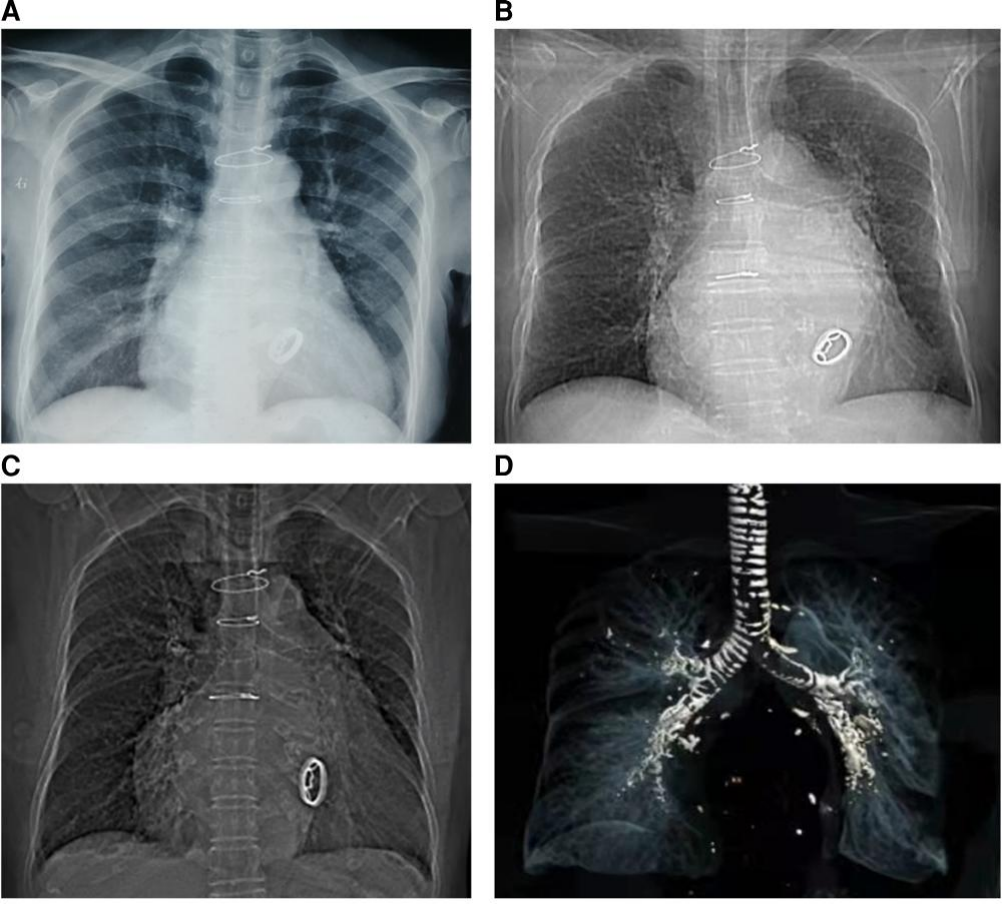
A series of chest X-ray indicated the progressive calcification in the tracheobronchial tree. (*A*) Sixteen years after mitral valve replacement, the chest radiography was unremarkable. (*B*) Ten years prior to admission, the chest radiography revealed a mild diffused tracheobronchial calcification. (*C*) Chest imaging at admission, 31 years after mitral valve replacement showed severe tracheobronchial calcification. (*D*) The remoulding of the tracheobronchial tree revealed diffused and severe tracheobronchial tree calcification.

**Figure 2:**
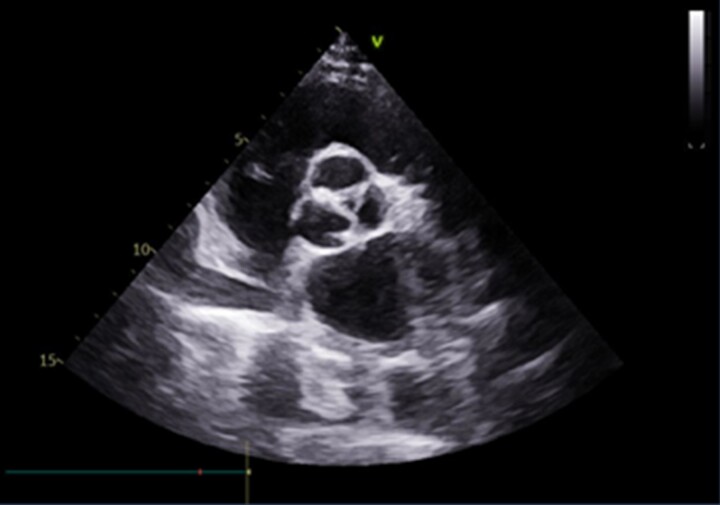
Tricuspid severely stenosed calcified aortic valve on echocardiographic examination.

**Figure 3:**
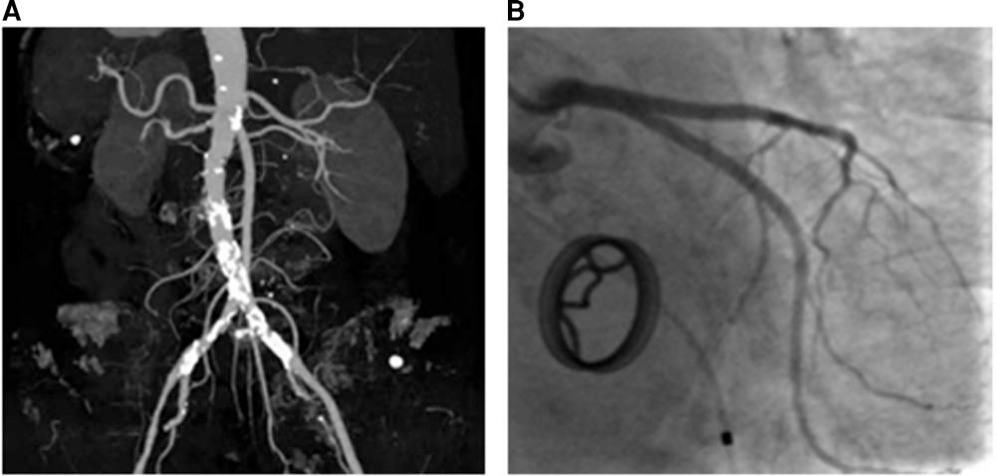
Diffused vascular calcification. (*A*) Calcification in the abdominal aorta. (*B*) Mild calcification in the left anterior descending artery.

**Figure 4:**
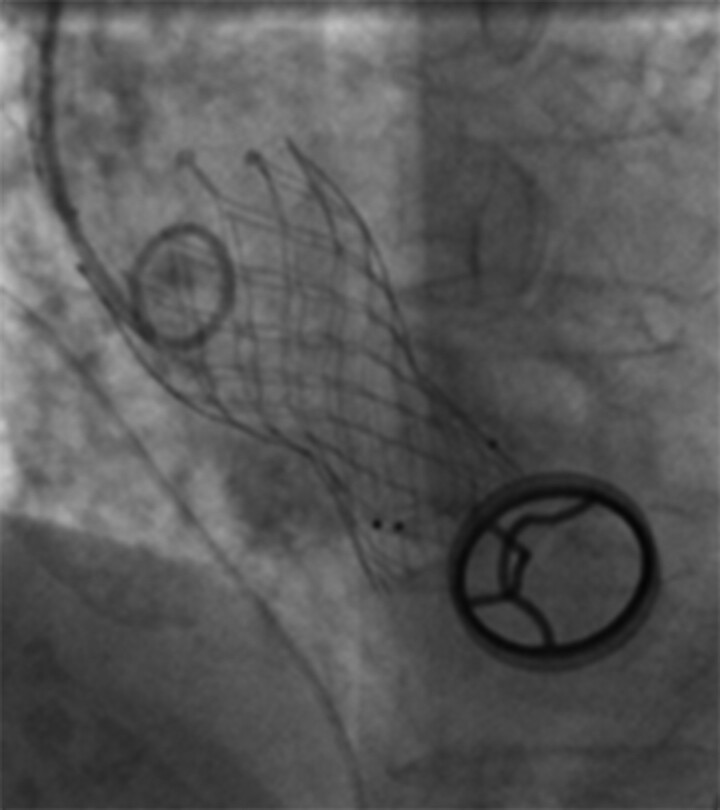
Transcatheter aortic valve replacement.

A 78-year-old woman was admitted with dyspnoea on exertion. When she was 46 years old, she was diagnosed with severe rheumatic mitral stenosis and received a tilting disc mechanical mitral valve (Size 26#, Shanghai made) implantation which required an optimal international normalized ratio (INR) between 2.0 and 3.0. The patient has been taking warfarin ever since, monitoring the INR level regularly during follow up and adjusting the dose of warfarin based on the INR value. However, she underwent three times of gastrointestinal haemorrhages when the INR exceeded 2.5, and based on some studies regarding the optimal INR level in Asian patients,^1,2^ an INR between 1.8 and 2.5 was therefore recommended, and the patient did not undergo episodes of stroke or systemic embolism. Thereafter, a routine echocardiography examination during follow up indicated a normal aortic valve and the mechanical mitral valve worked normally. A series of blood tests revealed the anti-streptolysin O titre was in the normal range. Sixteen years after mitral valve replacement, the chest radiography during her medical check-up was unremarkable (*Figure 1A*) and 5 years later, the chest radiography revealed a mild diffused tracheobronchial calcification (*Figure 1B*); however, she was asymptomatic. Twenty-five years after mitral valve replacement, the patient started feeling dyspnoea on exertion and swelling of both legs. Then echocardiography indicated a severe aortic valve calcification and stenosis with a valve orifice area of 0.7 cm^2^. She was suggested to receive a surgical or percutaneous aortic valve replacement, but she refused the intervention. From then on, she only received medical therapy including diuretics, beta-blockers, and a follow-up chest CT showed extensive calcification of the tracheobronchial rings, which was more significant than 5 years before. She was advised to seek the causes of calcification in the respiratory tract and aortic valve; however, there was no evidence of bone disease, thyroid abnormality, renal disease, autoimmune diseases, and serum calcium and phosphorus were normal. Moreover, the patient had no history of hypertension, dyslipidaemia, diabetes mellitus, coronary artery disease, etc. Due to the long-term use of warfarin, the association between warfarin with the calcification in the respiratory tract and aortic valve was suspected. One month prior to admission, her dyspnoea became worse, and therefore she was readmitted. Upon admission, she was hemodynamically stable with a heart rate of 72 beats per minute and blood pressure of 109/60 mmHg. Physical examination revealed mild bilateral ankle oedema and a systolic murmur in the aortic area. A chest imaging showed a progressive calcification of the tracheobronchial ring compared with before (*Figure 1C and D*). Echocardiographic examination revealed severe aortic stenosis with a valve orifice area of 0.5 cm^2^ (*Figure 2*). Moreover, a CT angiography showed diffused calcification in the abdominal aorta (*Figure 3A*) and mild calcification in the left anterior descending artery (*Figure 3B*). The patient was treated with warfarin 1.25 mg/day for anticoagulation therapy and furosemide 20–80 mg/day according to the urine volume to relieve symptoms of heart failure. Due to the severe aortic stenosis and symptom of heart failure, aortic valve replacement was recommended. The patient preferred to receive transcatheter aortic valve replacement because of the high risk of surgical intervention. A biologic aortic valve (Size L23, Venus, MedTech) was successfully implanted (*Figure 4*), and the symptom of dyspnoea and oedema significantly improved after transcatheter aortic valve replacement and the patient was discharged afterward. Medications after discharge included warfarin 1.25 mg/day for anticoagulation and furosemide 20 mg/day for diuresis. Six months post-aortic valve replacement, the patient remained free of exertional dyspnoea and oedema of the lower limbs.

## Discussion

In our case, we report a possible complication of long-term prophylactic anticoagulant therapy with warfarin, that is, diffused calcification. Warfarin is widely used to treat and prevent thromboembolic disease; however, long-term use of warfarin is associated with some potential side effects such as cholesterol microembolization,^3^ nephropathy,^4^ vascular calcification,^5^ osteoporosis,^6^ calciphylaxis,^7^ skin necrosis,^8^ and vasculitis,^9^ although the incidence of these complications is relatively rare.

In our case, the patient presented with diffused calcification in multiple sites including the aorta, coronary artery, aortic valve, and the tracheobronchial tree. The mechanisms involved in vascular calcification associated with warfarin are complex and not completely understood. However, a protein named matrix gamma-carboxyglutamate Gla protein (MGP)^10^ has been linked to the process of vascular calcification. MGP is a mineral-binding, extracellular matrix protein that is mainly secreted by chondrocytes and vascular smooth muscle cells in the arterial tunica media.^10^ Studies have shown that MGP plays an important role in inhibiting calcification through many pathways such as vesicle-related mechanisms,^6^ binding to calcium ions, inhibiting calcium crystal growth,^7^ preventing vascular smooth muscle cells from differentiating into osteogenic cells,^8^ regulating vascular smooth muscle cells apoptosis cycle,^9^ etc. The reason why long-term warfarin use is associated with increased vascular calcification is due to warfarin’s competitively inhibiting VKORC1, while the MGP’s inhibiting vascular calcification relies on the active form of vitamin K.^10^ Therefore, reduction of vitamin K activation decreases the number of MGP and leads to vascular calcification.

Aortic valve calcification is common in the elder and can cause aortic valve stenosis. In our case, in addition to senile degenerative change, the possibility of long-term warfarin-associated aortic valve stenosis cannot be ruled out. Previous studies have demonstrated that the use of warfarin was associated with an increased risk of valve calcification.^11^ Although the precise mechanism of calcification of the aortic valve is poorly understood, it seems the pathophysiological process likes that of atherosclerosis because they share some common cardiovascular risk factors such as hypertension, diabetes mellitus, dyslipidaemia, etc.^12^ However, in our present case, this patient had no aforementioned cardiovascular risk factors. Therefore, the possibility of these cardiovascular risk factors causing calcification of the aortic valve was relatively low, and long-term use of warfarin might be the reasonable interpretation. It is worth noting that this patient had rheumatic valve damage previously and rheumatic aortic valve stenosis was therefore suspected; however, there was no obvious aortic valve impairment during a long period of follow up after mitral valve replacement and the anti-streptolysin O titre was in the normal range, suggesting the aortic valve stenosis was probably non-rheumatic. Moreover, the congenital bicuspid aortic valve is a common cause of aortic stenosis; however, in our case, echocardiography revealed a tricuspid valve rather than a bicuspid valve. Therefore, the calcified stenotic aortic valve seemed to be associated with chronic warfarin use.

The remarkable feature of this case is the extensive calcification in the tracheobronchial tree. Tracheobronchial calcification is common among the elderly and can be caused by a series of diseases such as tracheopathia osteochondroplastica, polychondritis, and amyloidosis^13^; however, each of these diseases has its characteristics on the trachea imaging. Based on the medical history, chest imaging, and biochemical test, the aforementioned diseases can be excluded. Although the correlation between long-term warfarin use and progressive tracheobronchial calcification seems well established, however, only limited cases were reported.^13,14^ Our case presented a progressive tracheobronchial tree calcification resulting from long-term warfarin treatment. To date, the exact mechanism of how warfarin induced tracheobronchial tree calcification is unclear; however, previous studies proposed the same mechanism with vascular calcification, as tracheobronchial calcification has also been observed in the patient with Keutel syndrome, a genetic syndrome with mutations on the gene regulate the function of MGP protein.^15^ Although tracheobronchial calcification due to warfarin is usually diffused, it is always asymptomatic and is not a contraindication for continuing warfarin therapy.

## Conclusion

Our case showed a patient presented with aortic valve calcification and diffused aortic and tracheobronchial calcification potentially related to long-term warfarin use. Although there are many side effects associated with long-term warfarin use, patients with valvular atrial fibrillation or with mechanical valve replacement have no other choice for anticoagulant medication except warfarin. Therefore, close monitoring of such side effects in patients with long-term use of warfarin is warranted.

## Supplementary Material

ytac364_Supplementary_Data

## Lead author biography

Bryan Richard Sasmita, MBBS, was graduated from Chongqing Medical University in 2019. He is currently furthering his training and studies as a postgraduate student in the First Affiliated Hospital of Chongqing Medical University. He intends to take a PhD in cardiology.
