# Supplementary material for: Diffused calcification in a patient with long-term warfarin therapy: a case report
Source: Eur Heart J Case Rep. 2022 Sep 1;6(9):ytac364. doi: 10.1093/ehjcr/ytac364 (PMC9486598; doi:10.1093/ehjcr/ytac364)
Supplement: ytac364_Supplementary_Data [file ytac364_supplementary_data.zip › Revised Slide Set.pptx]

## Slide 1
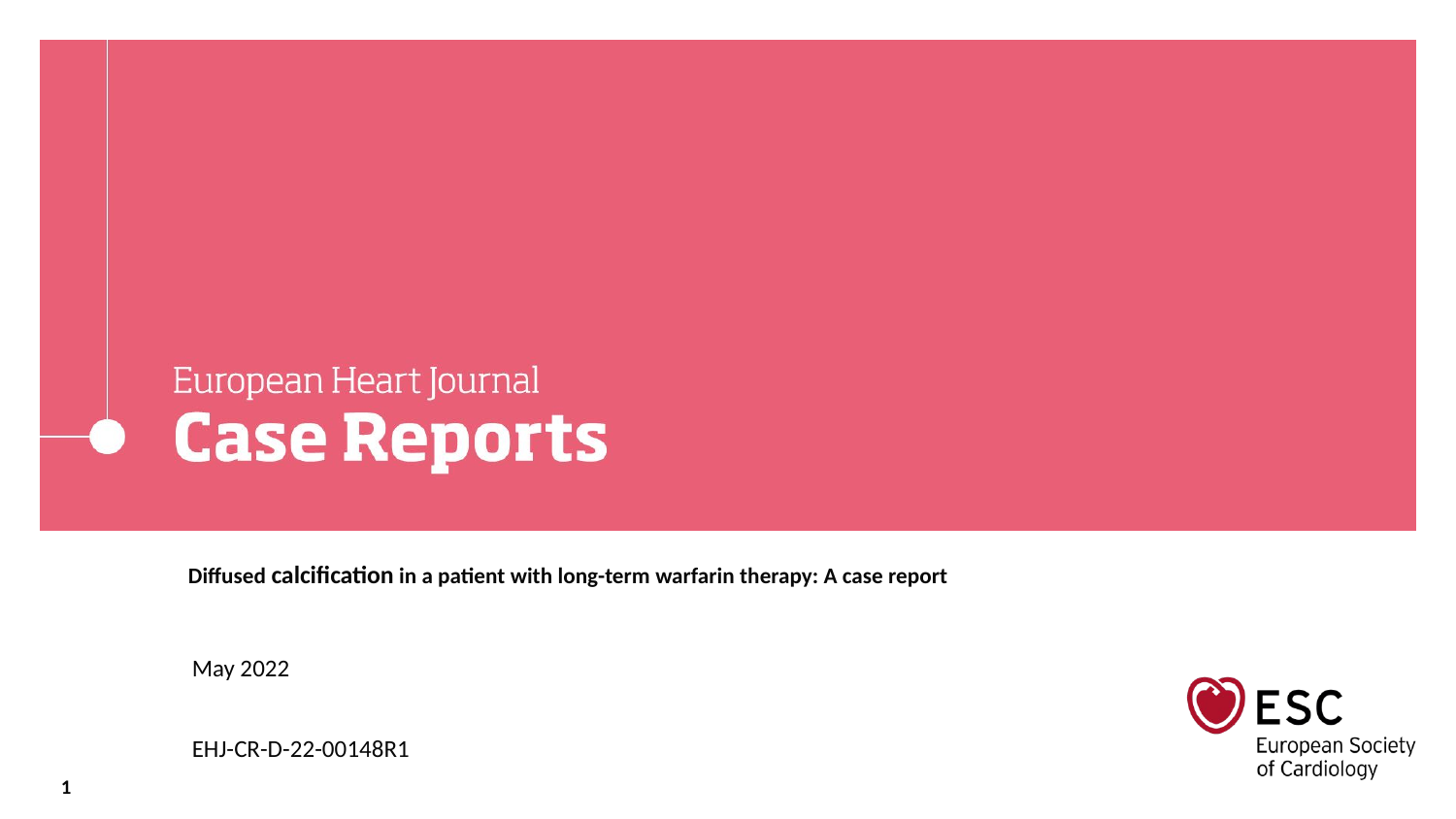

# Diffused calcification in a patient with long-term warfarin therapy: A case report
May 2022
EHJ-CR-D-22-00148R1
1

## Slide 2
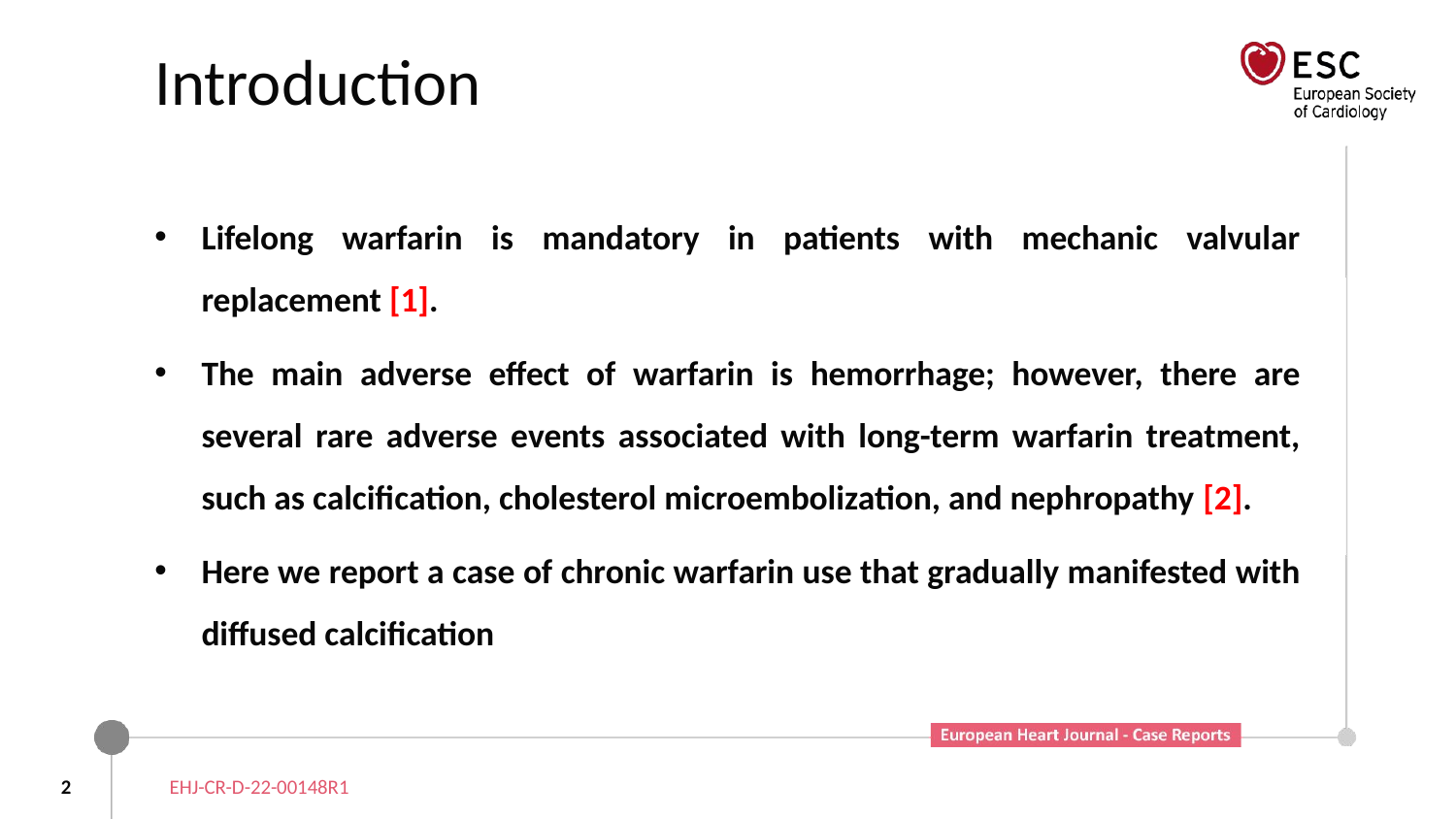

# Introduction
Lifelong warfarin is mandatory in patients with mechanic valvular replacement [1].
The main adverse effect of warfarin is hemorrhage; however, there are several rare adverse events associated with long-term warfarin treatment, such as calcification, cholesterol microembolization, and nephropathy [2].
Here we report a case of chronic warfarin use that gradually manifested with diffused calcification
2
EHJ-CR-D-22-00148R1

## Slide 3
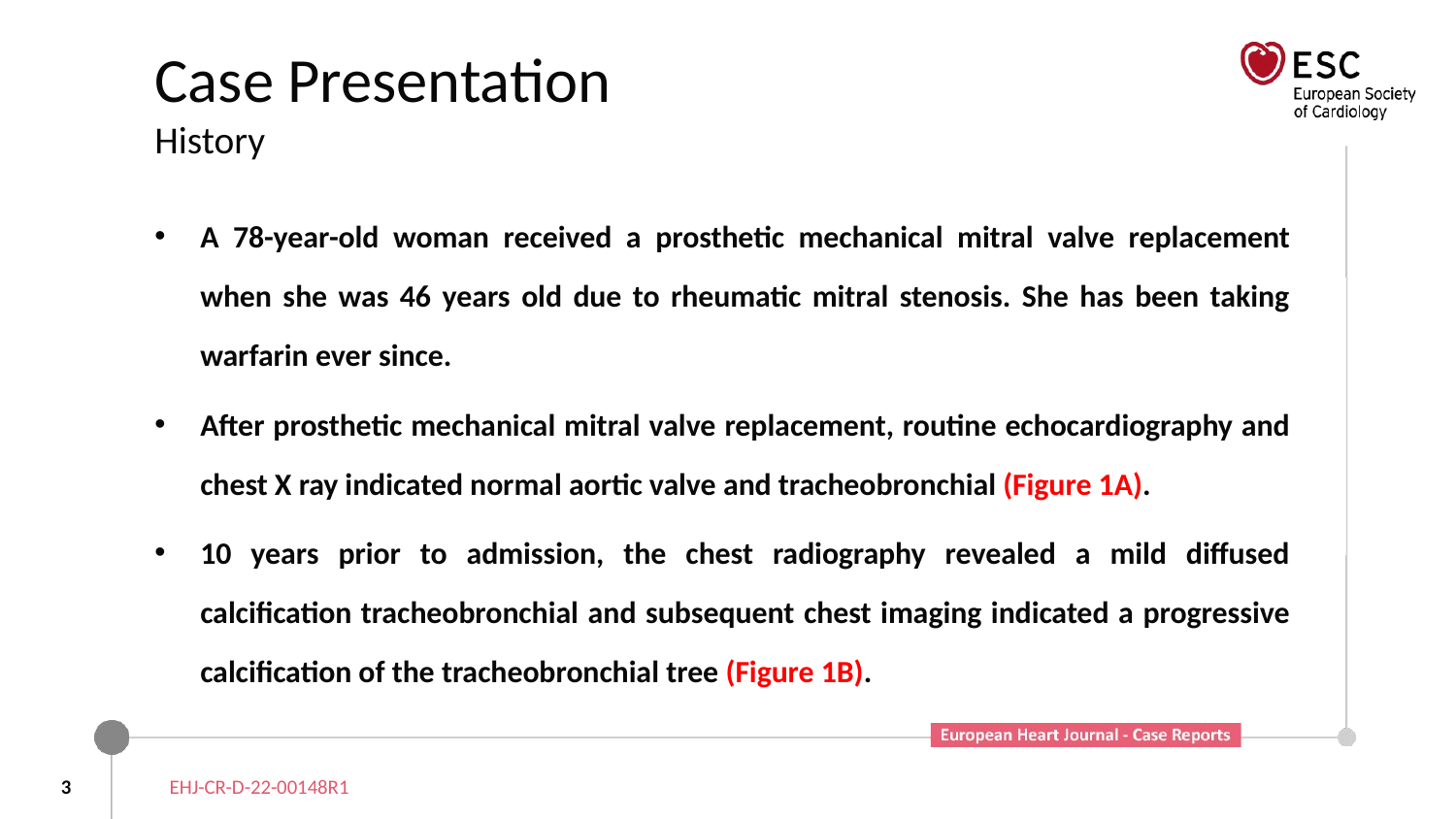

# Case Presentation History
A 78-year-old woman received a prosthetic mechanical mitral valve replacement when she was 46 years old due to rheumatic mitral stenosis. She has been taking warfarin ever since.
After prosthetic mechanical mitral valve replacement, routine echocardiography and chest X ray indicated normal aortic valve and tracheobronchial (Figure 1A).
10 years prior to admission, the chest radiography revealed a mild diffused calcification tracheobronchial and subsequent chest imaging indicated a progressive calcification of the tracheobronchial tree (Figure 1B).
3
EHJ-CR-D-22-00148R1

## Slide 4
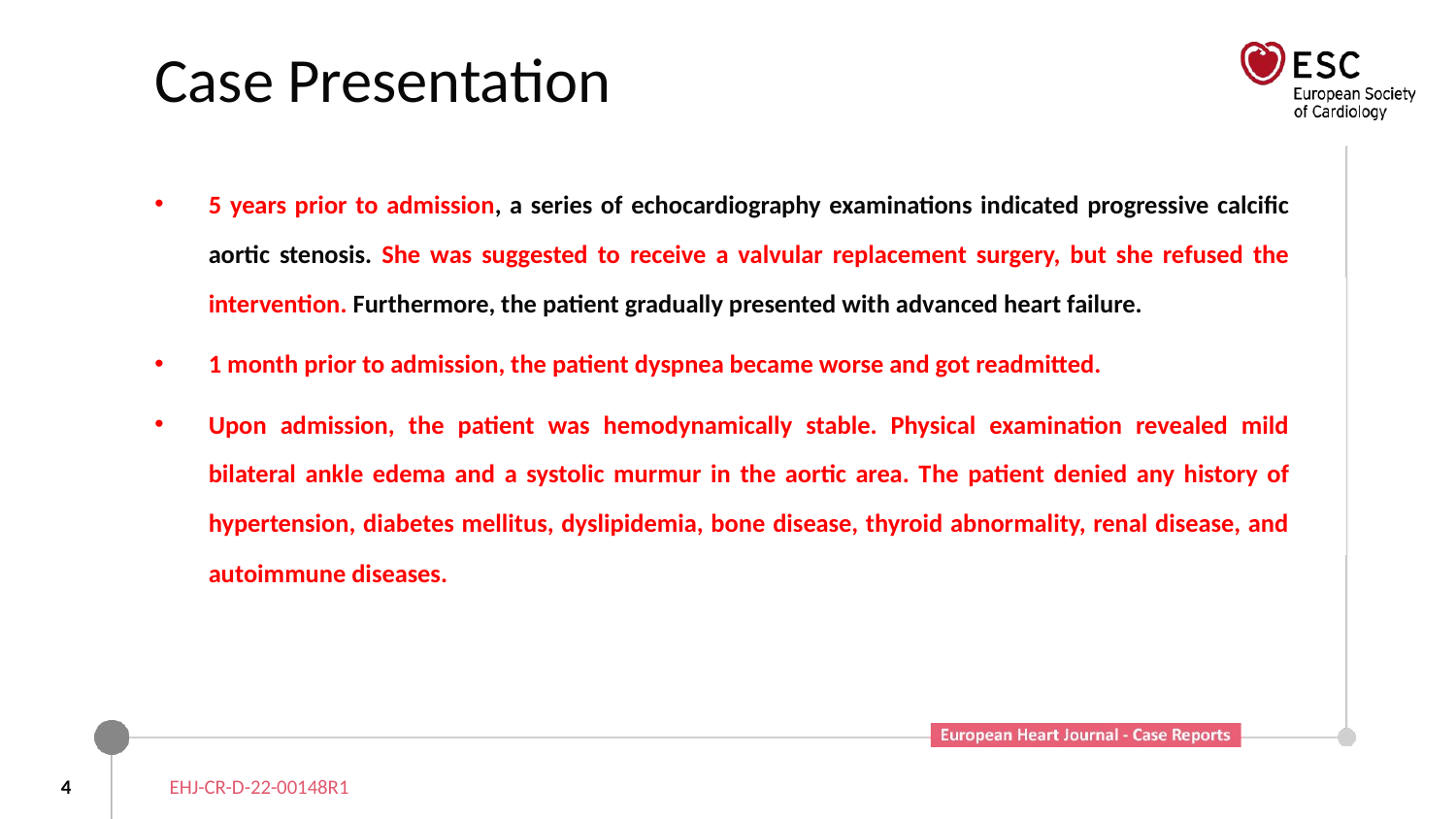

# Case Presentation
5 years prior to admission, a series of echocardiography examinations indicated progressive calcific aortic stenosis. She was suggested to receive a valvular replacement surgery, but she refused the intervention. Furthermore, the patient gradually presented with advanced heart failure.
1 month prior to admission, the patient dyspnea became worse and got readmitted.
Upon admission, the patient was hemodynamically stable. Physical examination revealed mild bilateral ankle edema and a systolic murmur in the aortic area. The patient denied any history of hypertension, diabetes mellitus, dyslipidemia, bone disease, thyroid abnormality, renal disease, and autoimmune diseases.
4
EHJ-CR-D-22-00148R1

## Slide 5
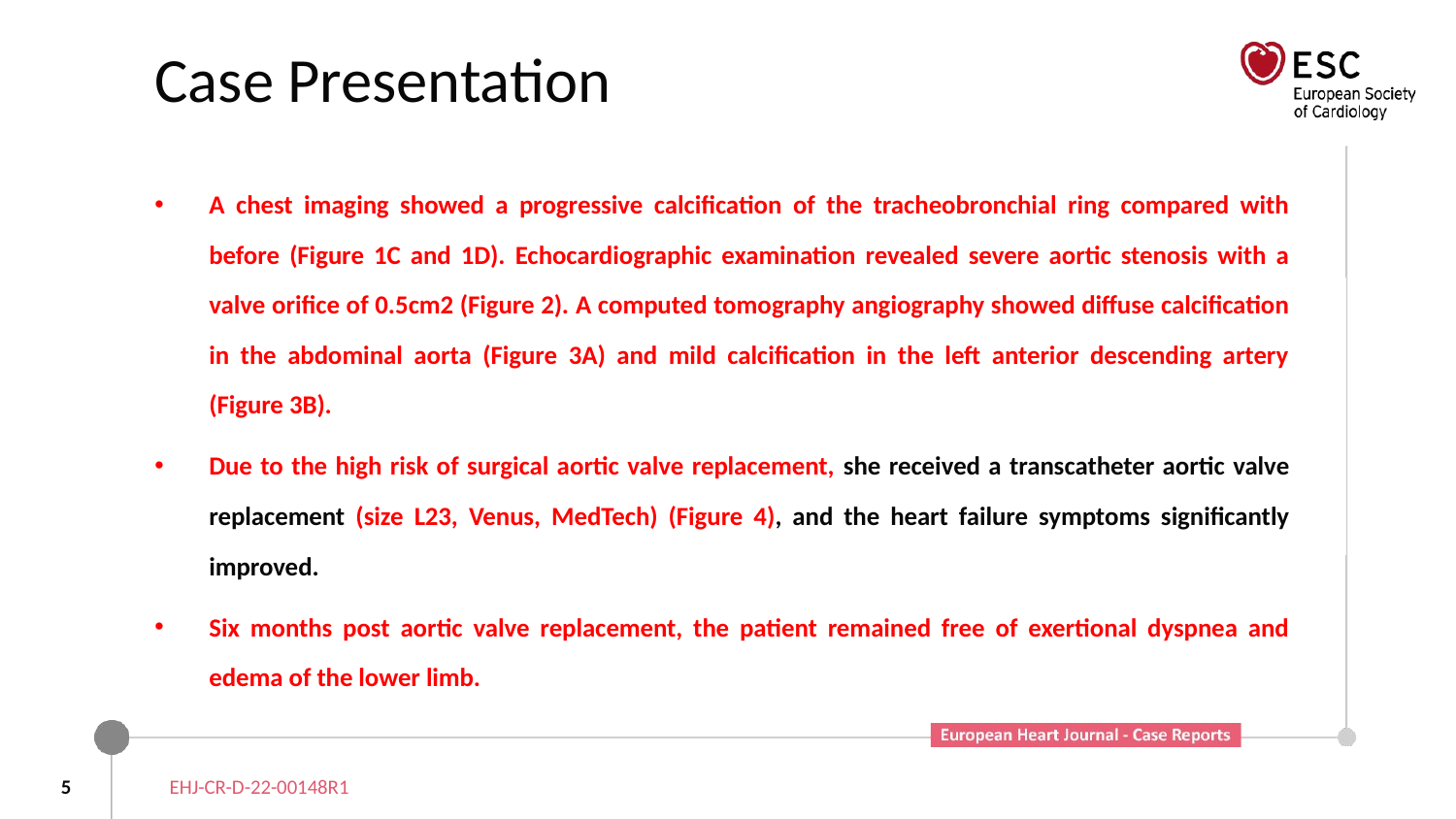

# Case Presentation
A chest imaging showed a progressive calcification of the tracheobronchial ring compared with before (Figure 1C and 1D). Echocardiographic examination revealed severe aortic stenosis with a valve orifice of 0.5cm2 (Figure 2). A computed tomography angiography showed diffuse calcification in the abdominal aorta (Figure 3A) and mild calcification in the left anterior descending artery (Figure 3B).
Due to the high risk of surgical aortic valve replacement, she received a transcatheter aortic valve replacement (size L23, Venus, MedTech) (Figure 4), and the heart failure symptoms significantly improved.
Six months post aortic valve replacement, the patient remained free of exertional dyspnea and edema of the lower limb.
5
EHJ-CR-D-22-00148R1

## Slide 6
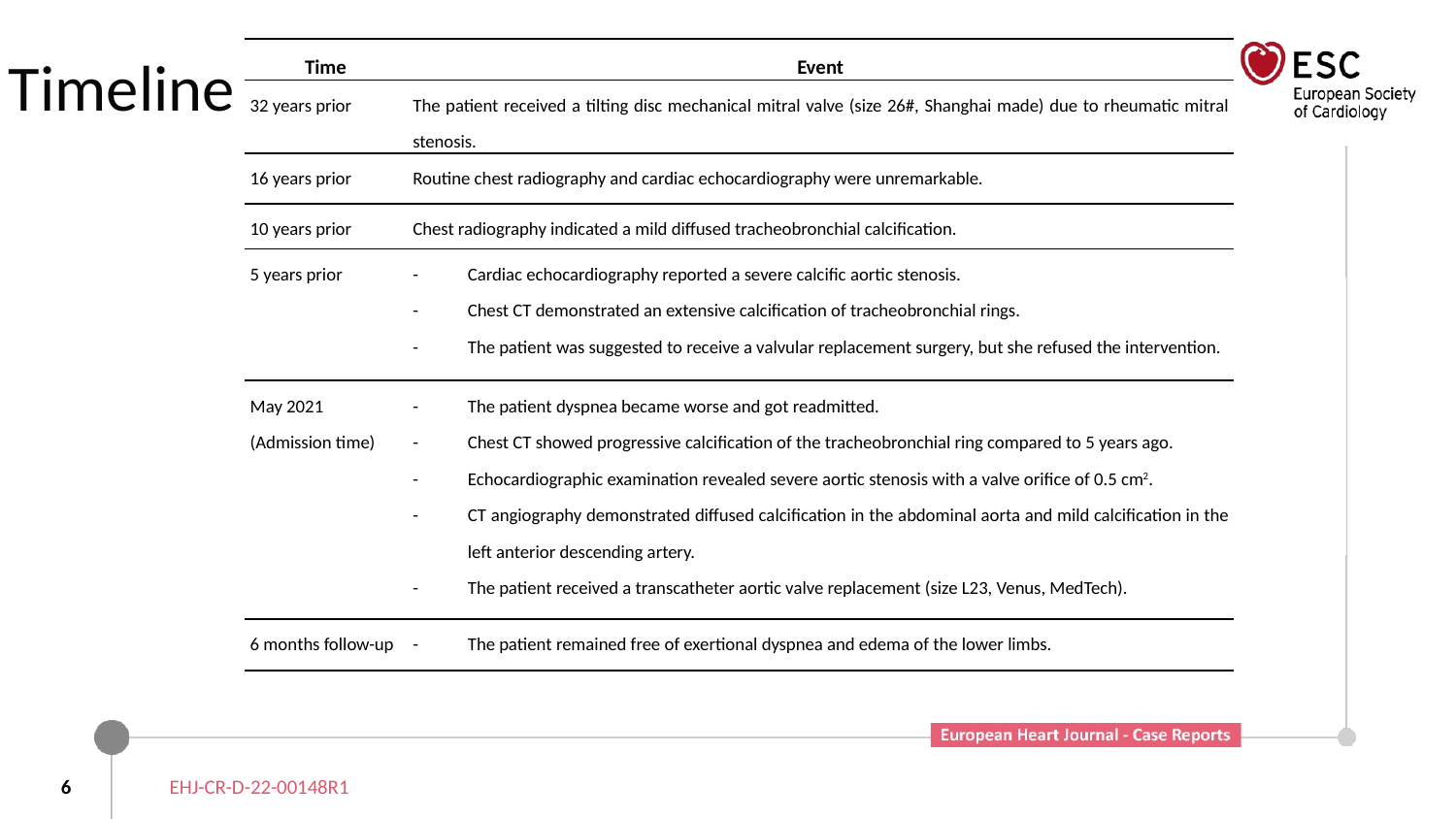

# Timeline
| Time | Event |
| --- | --- |
| 32 years prior | The patient received a tilting disc mechanical mitral valve (size 26#, Shanghai made) due to rheumatic mitral stenosis. |
| 16 years prior | Routine chest radiography and cardiac echocardiography were unremarkable. |
| 10 years prior | Chest radiography indicated a mild diffused tracheobronchial calcification. |
| 5 years prior | Cardiac echocardiography reported a severe calcific aortic stenosis. Chest CT demonstrated an extensive calcification of tracheobronchial rings. The patient was suggested to receive a valvular replacement surgery, but she refused the intervention. |
| May 2021 (Admission time) | The patient dyspnea became worse and got readmitted. Chest CT showed progressive calcification of the tracheobronchial ring compared to 5 years ago. Echocardiographic examination revealed severe aortic stenosis with a valve orifice of 0.5 cm2. CT angiography demonstrated diffused calcification in the abdominal aorta and mild calcification in the left anterior descending artery. The patient received a transcatheter aortic valve replacement (size L23, Venus, MedTech). |
| 6 months follow-up | The patient remained free of exertional dyspnea and edema of the lower limbs. |
6
EHJ-CR-D-22-00148R1

## Slide 7
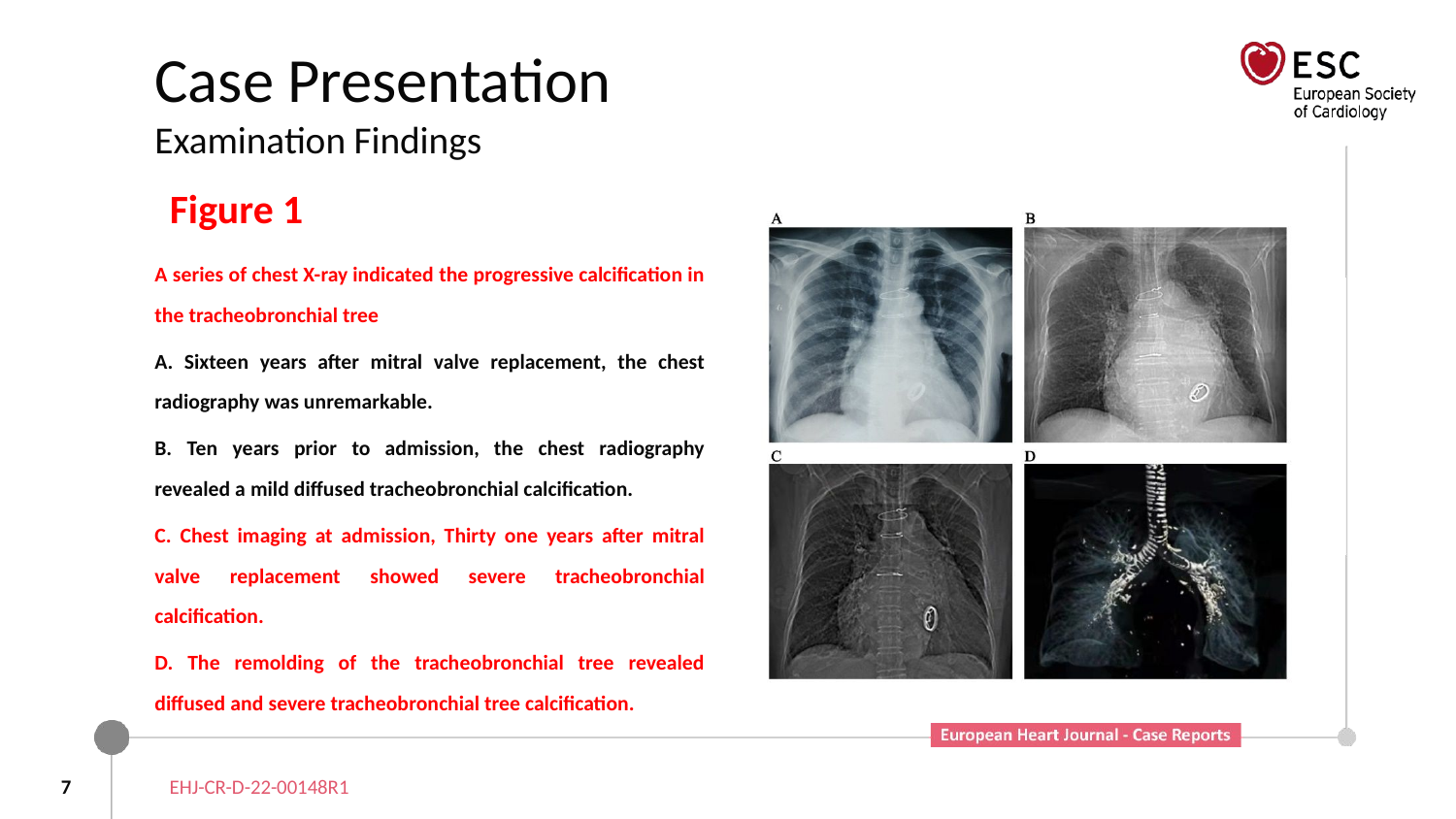

# Case Presentation Examination Findings
Figure 1
A series of chest X-ray indicated the progressive calcification in the tracheobronchial tree
A. Sixteen years after mitral valve replacement, the chest radiography was unremarkable.
B. Ten years prior to admission, the chest radiography revealed a mild diffused tracheobronchial calcification.
C. Chest imaging at admission, Thirty one years after mitral valve replacement showed severe tracheobronchial calcification.
D. The remolding of the tracheobronchial tree revealed diffused and severe tracheobronchial tree calcification.
7
EHJ-CR-D-22-00148R1

## Slide 8
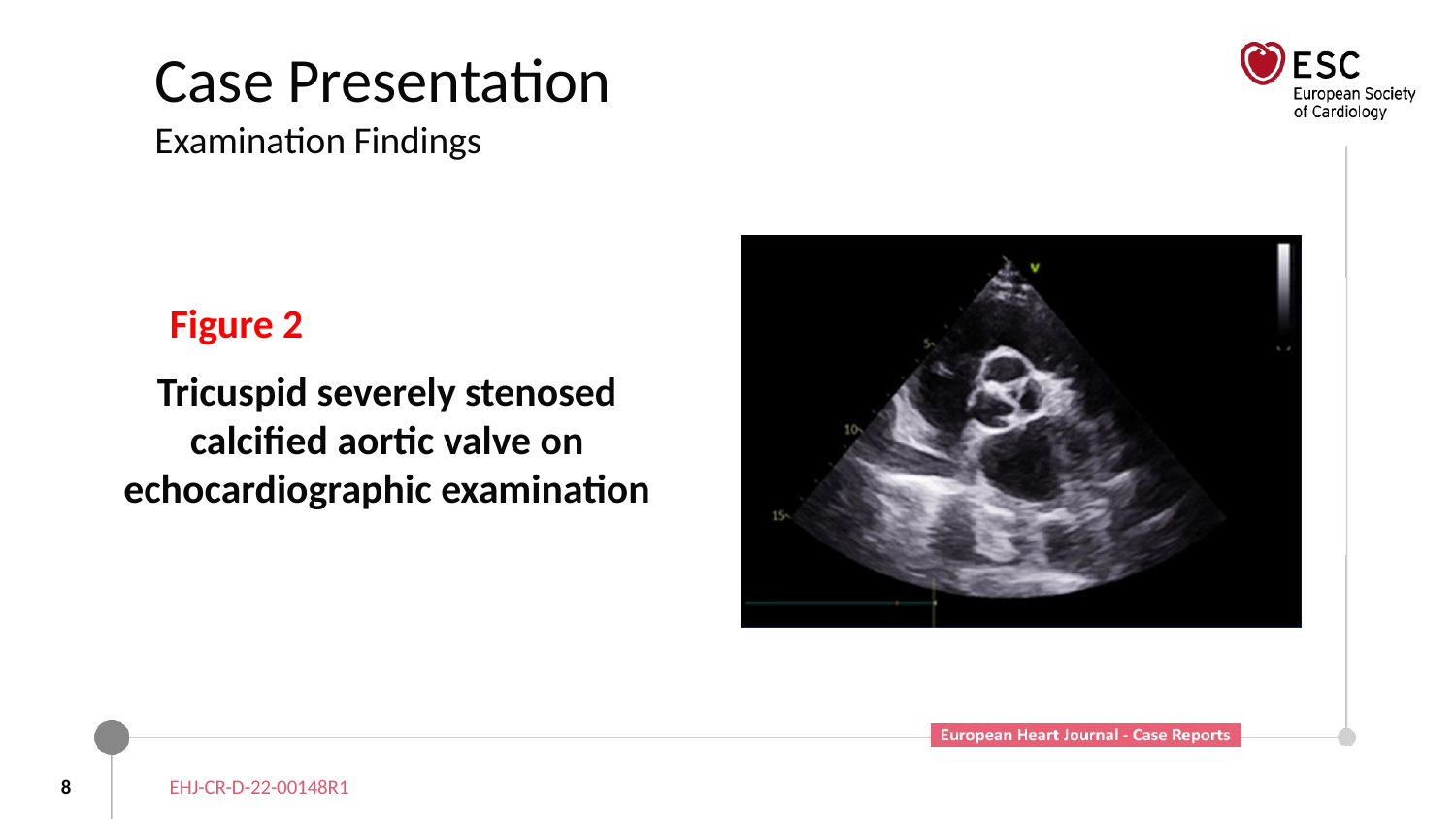

# Case Presentation Examination Findings
Tricuspid severely stenosed calcified aortic valve on echocardiographic examination
Figure 2
8
EHJ-CR-D-22-00148R1

## Slide 9
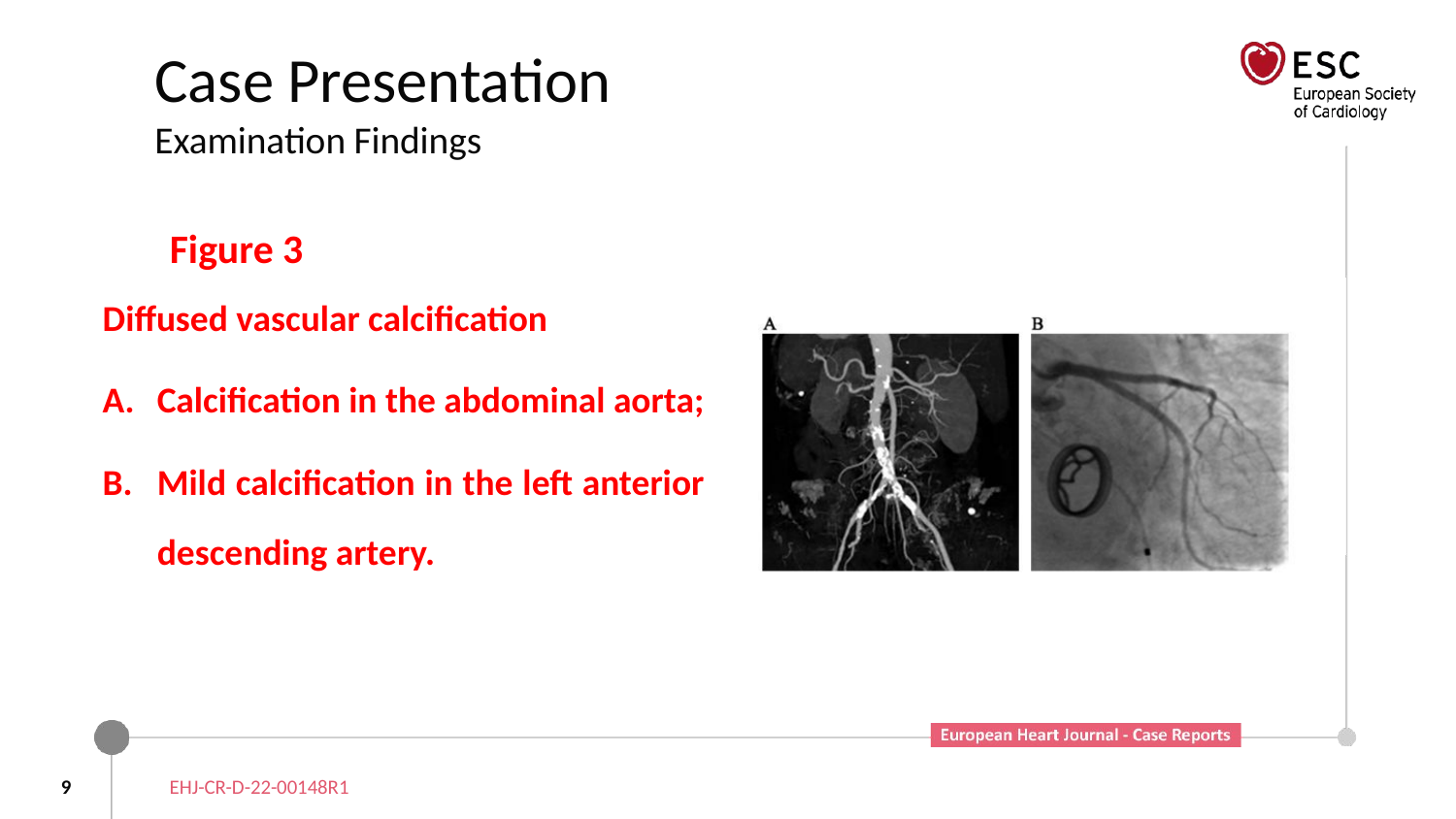

# Case Presentation Examination Findings
Figure 3
Diffused vascular calcification
Calcification in the abdominal aorta;
Mild calcification in the left anterior descending artery.
9
EHJ-CR-D-22-00148R1

## Slide 10
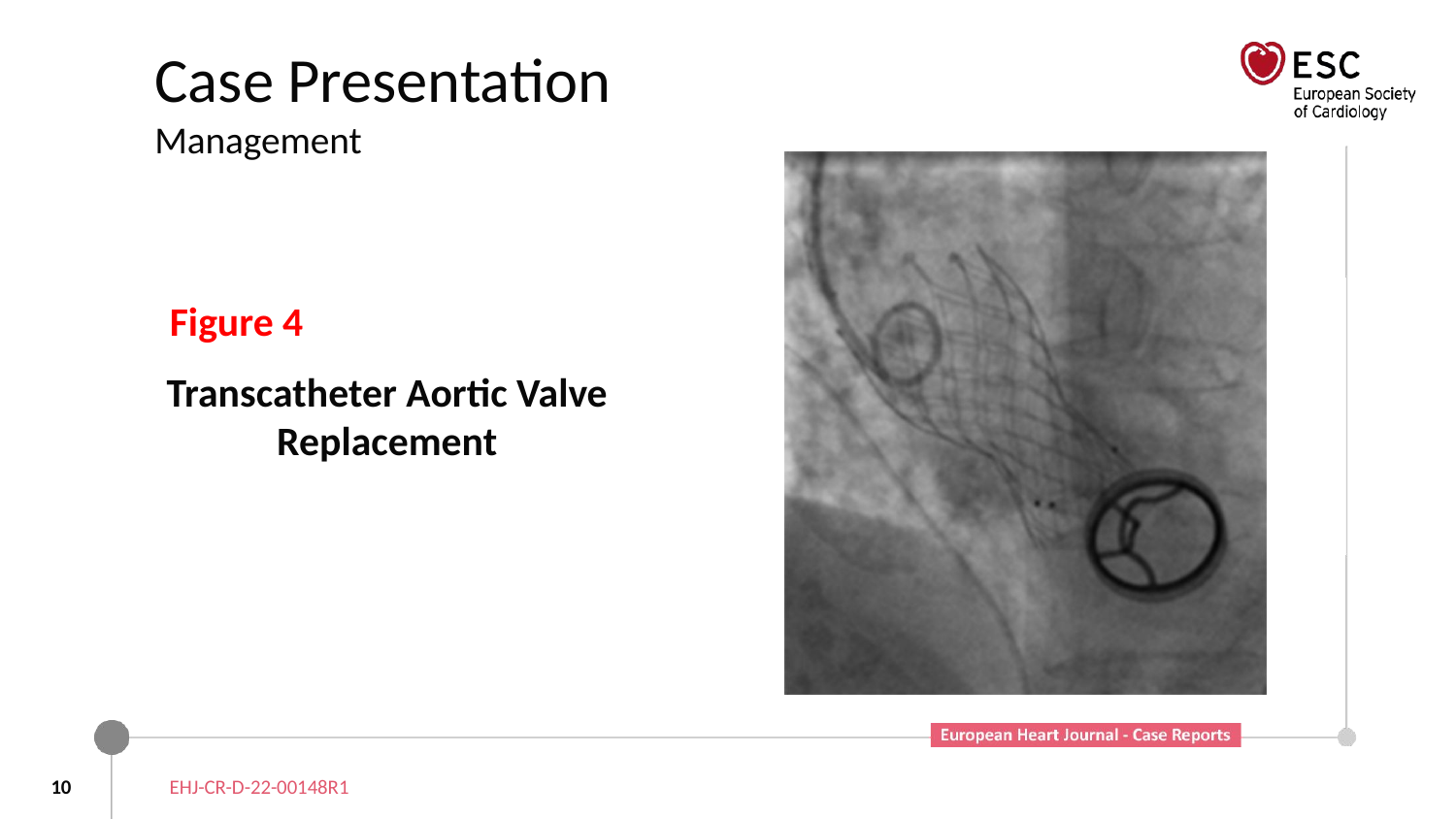

# Case Presentation Management
Transcatheter Aortic Valve Replacement
Figure 4
10
EHJ-CR-D-22-00148R1

## Slide 11
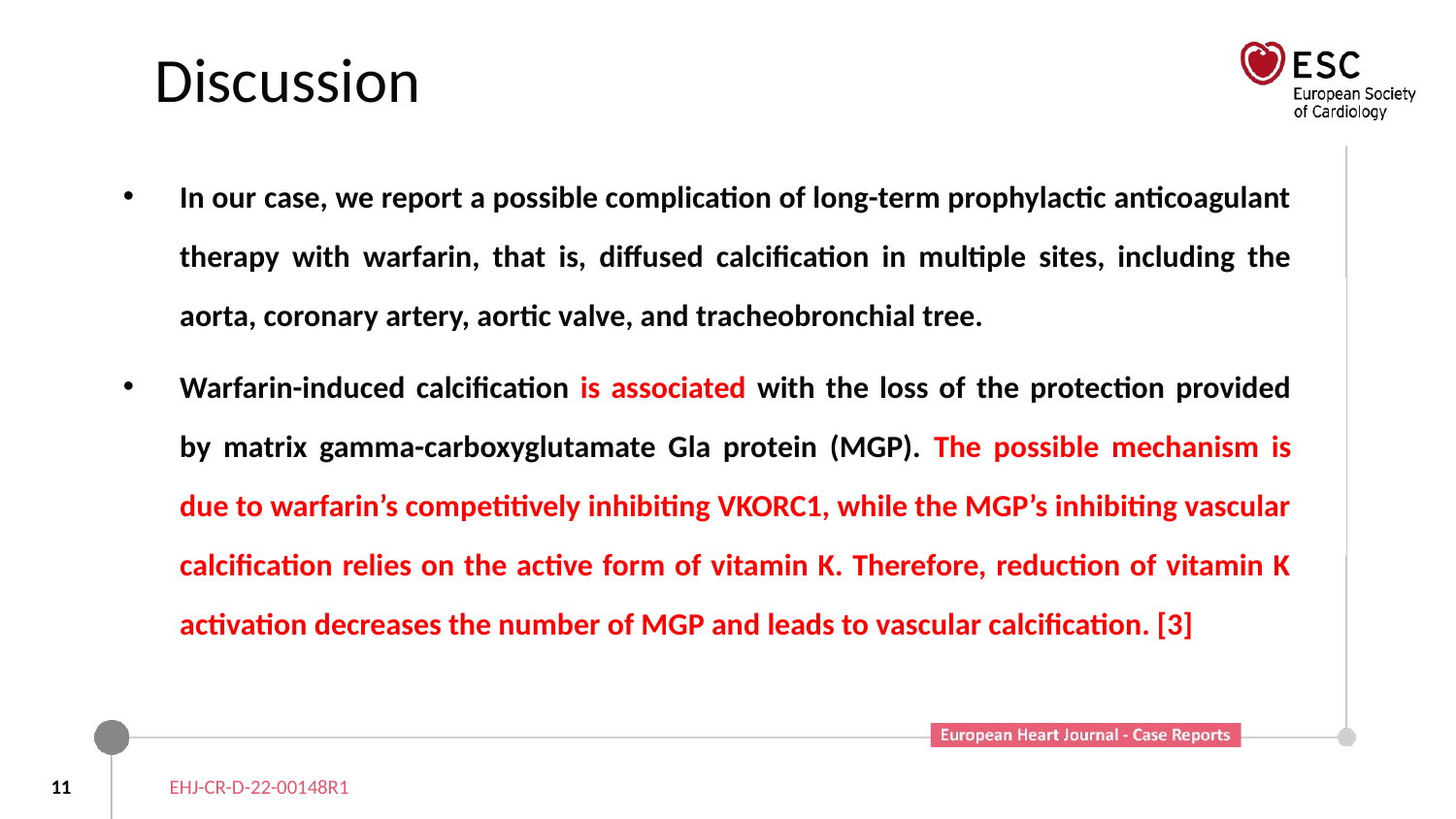

# Discussion
In our case, we report a possible complication of long-term prophylactic anticoagulant therapy with warfarin, that is, diffused calcification in multiple sites, including the aorta, coronary artery, aortic valve, and tracheobronchial tree.
Warfarin-induced calcification is associated with the loss of the protection provided by matrix gamma-carboxyglutamate Gla protein (MGP). The possible mechanism is due to warfarin’s competitively inhibiting VKORC1, while the MGP’s inhibiting vascular calcification relies on the active form of vitamin K. Therefore, reduction of vitamin K activation decreases the number of MGP and leads to vascular calcification. [3]
11
EHJ-CR-D-22-00148R1

## Slide 12
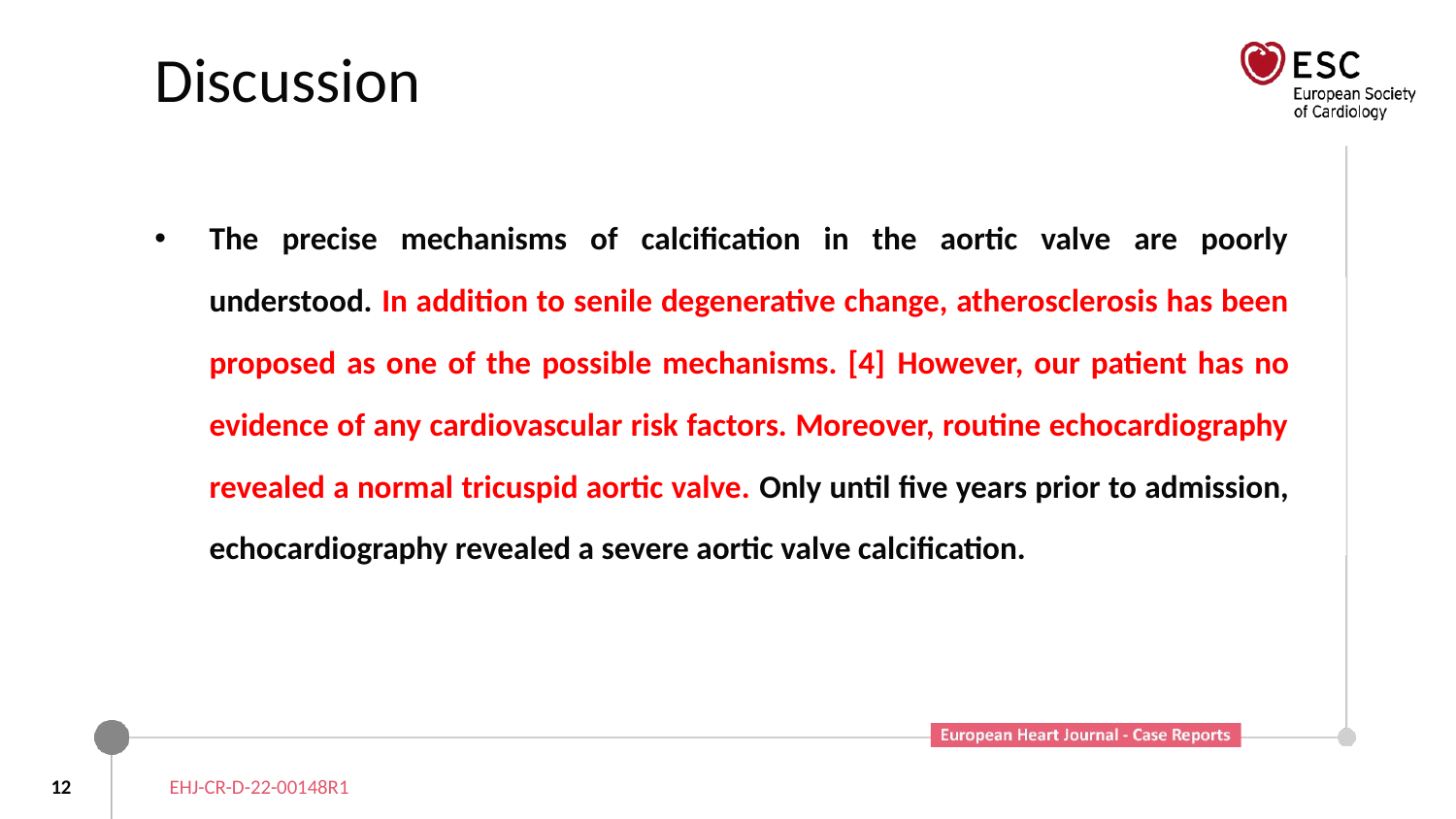

# Discussion
The precise mechanisms of calcification in the aortic valve are poorly understood. In addition to senile degenerative change, atherosclerosis has been proposed as one of the possible mechanisms. [4] However, our patient has no evidence of any cardiovascular risk factors. Moreover, routine echocardiography revealed a normal tricuspid aortic valve. Only until five years prior to admission, echocardiography revealed a severe aortic valve calcification.
12
EHJ-CR-D-22-00148R1

## Slide 13
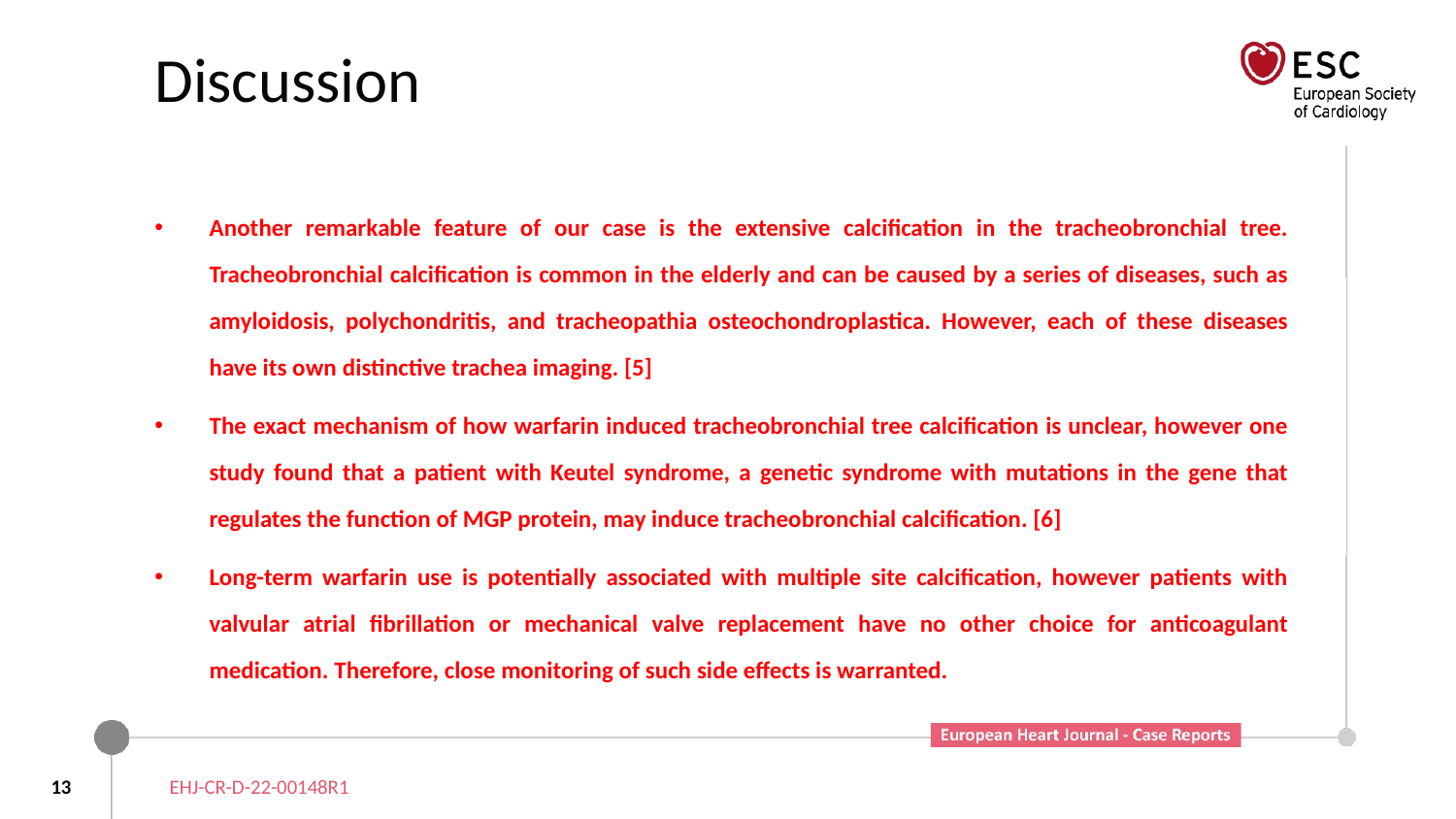

# Discussion
Another remarkable feature of our case is the extensive calcification in the tracheobronchial tree. Tracheobronchial calcification is common in the elderly and can be caused by a series of diseases, such as amyloidosis, polychondritis, and tracheopathia osteochondroplastica. However, each of these diseases have its own distinctive trachea imaging. [5]
The exact mechanism of how warfarin induced tracheobronchial tree calcification is unclear, however one study found that a patient with Keutel syndrome, a genetic syndrome with mutations in the gene that regulates the function of MGP protein, may induce tracheobronchial calcification. [6]
Long-term warfarin use is potentially associated with multiple site calcification, however patients with valvular atrial fibrillation or mechanical valve replacement have no other choice for anticoagulant medication. Therefore, close monitoring of such side effects is warranted.
13
EHJ-CR-D-22-00148R1

## Slide 14
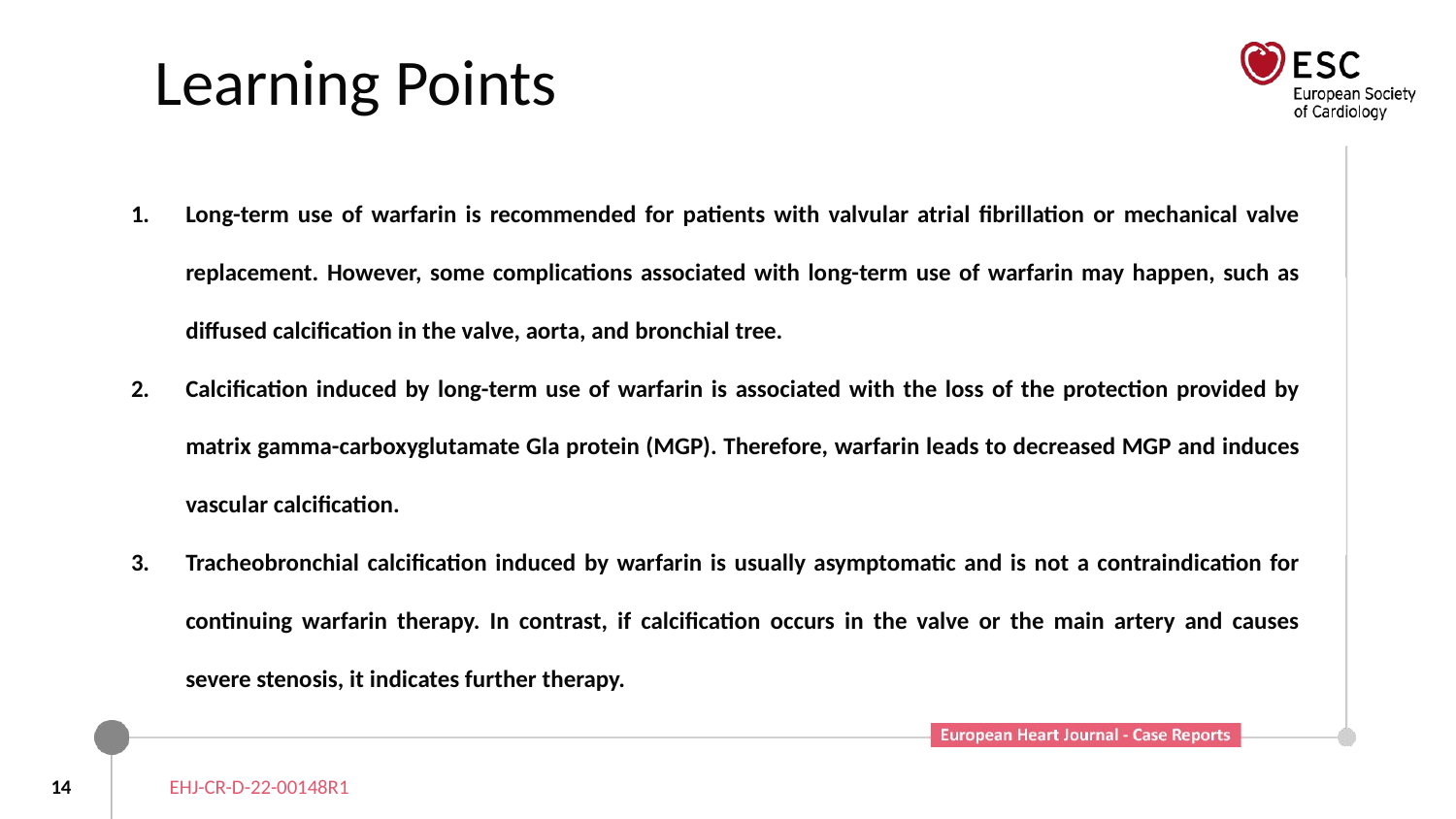

# Learning Points
Long-term use of warfarin is recommended for patients with valvular atrial fibrillation or mechanical valve replacement. However, some complications associated with long-term use of warfarin may happen, such as diffused calcification in the valve, aorta, and bronchial tree.
Calcification induced by long-term use of warfarin is associated with the loss of the protection provided by matrix gamma-carboxyglutamate Gla protein (MGP). Therefore, warfarin leads to decreased MGP and induces vascular calcification.
Tracheobronchial calcification induced by warfarin is usually asymptomatic and is not a contraindication for continuing warfarin therapy. In contrast, if calcification occurs in the valve or the main artery and causes severe stenosis, it indicates further therapy.
14
EHJ-CR-D-22-00148R1

## Slide 15
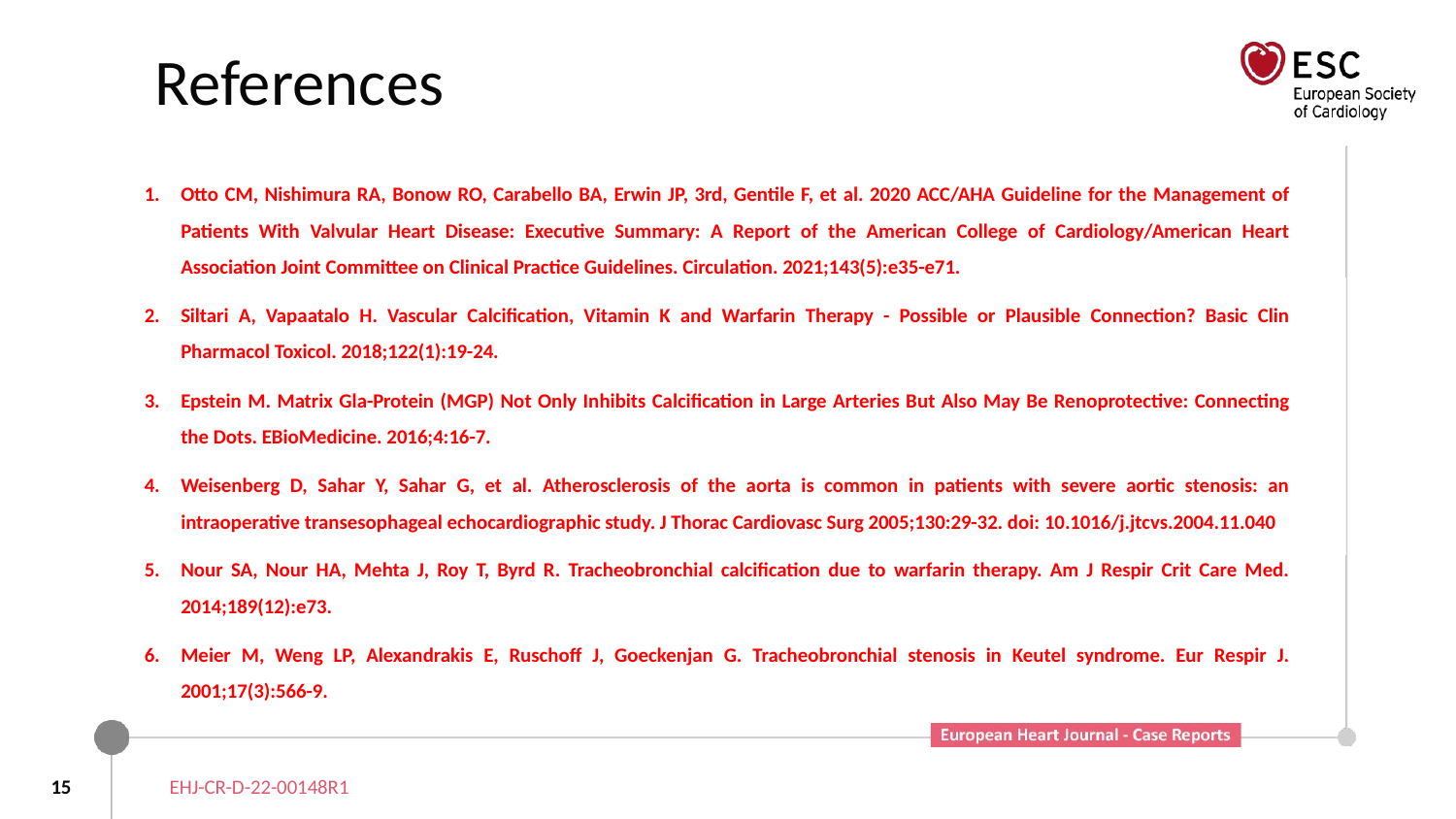

# References
Otto CM, Nishimura RA, Bonow RO, Carabello BA, Erwin JP, 3rd, Gentile F, et al. 2020 ACC/AHA Guideline for the Management of Patients With Valvular Heart Disease: Executive Summary: A Report of the American College of Cardiology/American Heart Association Joint Committee on Clinical Practice Guidelines. Circulation. 2021;143(5):e35-e71.
Siltari A, Vapaatalo H. Vascular Calcification, Vitamin K and Warfarin Therapy - Possible or Plausible Connection? Basic Clin Pharmacol Toxicol. 2018;122(1):19-24.
Epstein M. Matrix Gla-Protein (MGP) Not Only Inhibits Calcification in Large Arteries But Also May Be Renoprotective: Connecting the Dots. EBioMedicine. 2016;4:16-7.
Weisenberg D, Sahar Y, Sahar G, et al. Atherosclerosis of the aorta is common in patients with severe aortic stenosis: an intraoperative transesophageal echocardiographic study. J Thorac Cardiovasc Surg 2005;130:29-32. doi: 10.1016/j.jtcvs.2004.11.040
Nour SA, Nour HA, Mehta J, Roy T, Byrd R. Tracheobronchial calcification due to warfarin therapy. Am J Respir Crit Care Med. 2014;189(12):e73.
Meier M, Weng LP, Alexandrakis E, Ruschoff J, Goeckenjan G. Tracheobronchial stenosis in Keutel syndrome. Eur Respir J. 2001;17(3):566-9.
15
EHJ-CR-D-22-00148R1
